# Performance and challenges of power sector reform in China since 2015

**DOI:** 10.1016/j.isci.2025.113461

**Published:** 2025-08-28

**Authors:** Yanbo Feng, Jiang Lin, Feng Song, Mun Sing Ho

**Affiliations:** 1 School of Applied Economics, Renmin University of China, Beijing, China; 2 Department of Agricultural and Resources Economics, University of California, Berkeley, Berkeley, CA, USA; 3 Harvard-China Project on Energy, Economy and Environment, Harvard University, Cambridge, MA, USA

**Keywords:** Engineering, Electrical engineering, Energy sustainability

## Abstract

China has launched a comprehensive market-oriented reform of the power sector since 2015 to foster competition and enhance efficiency. This article summarizes the progress, achievements, and challenges of these reforms. Progress has been made in enhancing competition in wholesale and retail markets, strengthening regulation over grid costs and services, expanding inter-regional transmission, and promoting renewable electricity. Reform efforts have yielded several notable achievements: significantly improving generation efficiency, particularly among smaller-capacity units in the short term; substantially reducing industrial and commercial electricity prices, though primarily due to government intervention rather than market competition; effectively capturing market dynamics through spot market, although their limited scale has constrained broader impacts; and improving efficiency and welfare through market integration but raising concerns about equity. In the transition to a low-carbon energy future, two pivotal challenges remain: striking a balance between market mechanisms and government intervention; and ensuring system flexibility and long-term resource adequacy.

## Highlights

• The power market reform has enhanced generation efficiency
• Small-capacity generators have gained the most efficiency improvement
• The decline in electricity prices was mainly driven by strengthened regulation
• Spot markets effectively capture market dynamics

## Graphical abstract

**Figure:**
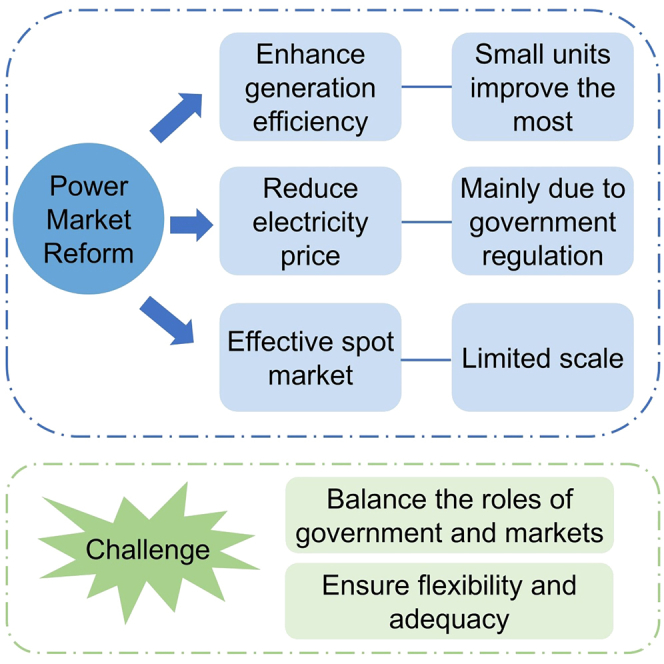

## Introduction

Historically, the great majority of electricity customers in most countries were served by a vertically integrated (and in some cases, government-owned) monopoly utility that provided generation, transmission, local distribution, and billing/collections. Since the 1980s, however, many countries have pursued power sector marketization and restructuring initiatives. Electricity market reforms seek to restructure vertically integrated power companies to improve their efficiency through market competition and encourage private sector participation in the power business.^1^

China has the world’s largest electricity sector, with 2.92 million megawatts (MW) of installed capacity and 9.22 billion megawatt-hours (MWh) of electricity consumption in 2023.^2^ In 2002, China initiated its first round of power sector reform, splitting the State Power Company and establishing two major power grid operators and five major power generation companies.^3^^,^^4^ The power grid operators would purchase electricity from power plants owned by the power generation companies at wholesale prices and then sell it to power users at government-guided retail prices.^5^ Although the 2002 reforms yielded considerable achievements, China’s power sector still faced critical challenges such as insufficient competition on the generation side, inadequate market-based pricing mechanisms, an underdeveloped regulatory system, and obstacles to renewable energy expansion.

To address these issues and advance power sector marketization, China launched a second round of reforms in 2015. These reforms fostered price competition in generation and retail, enhanced the regulation of grid costs and services, expanded inter-regional transmission, and promoted renewable electricity.^6^ To date, these reforms have achieved several impressive outcomes.^7^ Notably, China’s wholesale and retail electricity markets have been established, its transmission and distribution (T&D) pricing system has gradually formed, the role of the market in determining resource allocation has been significantly enhanced, and the proportion of electricity traded in the market has increased significantly.

Today, a decade after the 2015 reforms, China’s power sector has reached a critical juncture. Conducting a comprehensive review of progress, identifying unresolved issues, and charting a course for the future are essential to ensuring continued success. This study combines qualitative and quantitative methods to evaluate the reform’s achievements while identifying areas for improvement. Key conclusions include the following: the shift from “fairness-based” to market-based dispatch has significantly enhanced generation efficiency; small-capacity generators have gained the most efficiency improvement in the short term, but are unlikely to avoid eventual phase-out in the long run; industrial and commercial (I&C) electricity prices have seen notable declines, largely driven by enhanced regulatory supervision, especially in T&D, rather than by intensified market competition; and spot markets have effectively captured market dynamics, but their limited scale restricts broader impact. Looking ahead, China’s electricity reform faces two major challenges. First, balancing the roles of government and the market remains a complex task, as state involvement is vital for regulation, planning, and addressing market failures. Second, ensuring system flexibility and long-term adequacy is increasingly critical, particularly with the growing share of renewable energy.

The remainder of this article is organized as follows. In the section international experience with electricity market reform, we review global lessons from electricity sector reform. In the section progress of China’s electricity reform, we summarize China’s reform progress since 2015. In the section electricity market performance since the 2015 reforms, we assess the reforms from the perspectives of efficiency, price, and integration. In the section future challenges of China’s electricity market reform, we summarize the challenges currently faced by market reform efforts, as well as potential solutions. We conclude by summarizing China’s electricity market reform and offering a perspective on its future.

## Results

### International experience with electricity market reform

The electricity industry has four main segments: generation, transmission, distribution, and retailing/billing. Historically, all segments were vertically integrated within monopoly utility companies that served customers under “cost-of-service” regulation. These utilities were subject to the government regulation of prices, entry, investment, service quality, and other aspects of firm behavior.^8^^,^^9^ Since the 1980s, countries such as the U.S. and the UK have begun restructuring their power sectors to enable market competition and greater efficiency.^10^ Several studies have summarized the experience of electricity reforms around the world and distilled a theoretical basis and common principles.^11^^,^^12^^,^^13^ Crucially, market rules must create economic incentives for actions by individual market participants that enhance, or at least do not detract from, real-time system reliability or long-term supply adequacy. While China’s electricity market reform is shaped by its distinctive national conditions, it fundamentally adheres to similar principles and shares substantial commonalities with international reform practices.

#### Market restructuring and ownership changes

The restructuring of the power industry includes both vertical unbundling and horizontal restructuring. Vertical unbundling separates generation and retailing activities from the natural monopoly T&D segments to limit monopoly power.^14^^,^^15^ Horizontal restructuring increases the number of participants in wholesale and retail markets.^16^

##### Vertical unbundling to separate functions

Structural or functional vertical separation of competitive segments (i.e., generation, marketing, and retail supply) from regulated segments (i.e., distribution, transmission, and system operations) is viewed as essential to electricity market restructuring for two reasons. First, each segment exhibits different cost structures and innovation characteristics, leading to distinct optimal scales, risk profiles, and operational logics for each segment. Second, vertical unbundling can guard against the cross-subsidization of competitive businesses from regulated businesses, as well as discriminatory policies affecting competitive suppliers’ access to distribution and transmission networks.^10^

##### Horizontal restructuring to enhance competition

Horizontal restructuring increases the number of enterprises in competitive segments to mitigate market power and ensure reasonably competitive outcomes.^10^^,^^16^ In the generation segment, this typically involves breaking up existing firms and encouraging entry by new players. Both are done to avoid the establishment of dominant firms and ensure an economically efficient resource mix. Firms with an excessively large market share can use market power to raise prices, particularly during periods of scarcity.^7^ On the retail side, restructuring relaxes utilities’ monopoly franchise rights and promotes “customer choice” as a guiding principle. Consumers gain the flexibility to purchase electricity from competing suppliers, who either source power from wholesale markets or operate generation assets to fulfill retail supply obligations.^7^^,^^11^

In horizontal unbundling, large amounts of generation capacity were converted from monopoly utility or government ownership to private, competitive ownership. Most electricity reform models attach considerable importance and expectations to market-oriented reforms based on competition among non-monopoly private sector entities. Where private ownership of generation assets is not possible, reform efforts require power companies to operate on commercial principles. These principles require that enterprises pay taxes and market-based interest rates, earn commercially competitive returns on equity capital, and manage their own budgets, borrowing, procurement, and labor.^17^

#### Mechanisms to achieve real-time balance

Maintaining the stability of power systems requires the real-time balancing of electricity supply and demand. Prior to restructuring, regulated utilities centrally managed supply-demand coordination. The advent of market-oriented reforms introduced the need for mechanisms to align market participants’ actions with real-time balancing objectives. This need has become even more critical with the growth of variable renewable energy sources such as wind and solar, which require sophisticated solutions to ensure system reliability.

##### Short-term markets

Short-term markets reward generators for matching supply and demand in near real-time, which typically involves both day-ahead and intra-day (balancing) markets. This gives incentives to generators (and to loads) to adjust their position based on the general condition of the system and their own operating situation. Prices in short-term markets are often determined by marginal costs, where the price of electricity reflects the cost of the last unit of generation dispatched to meet demand. Short-term prices can provide transparent and constantly updated information and serve as the basis for determining longer-term contract prices. They also signal to the demand side of the market the value of short-run actions to reduce (or increase) power demand.

##### Ancillary service markets

The key attributes of real-time supply-demand balancing in electricity, along with other physical characteristics of electricity, significantly influence whether and how competition can be introduced. Electricity demand varies greatly across hours of the day and days of the year, and despite advances in storage technologies, cost-effective electricity storage remains a persistent challenge for both consumers and distributors. Consequently, the generation and consumption of electricity must be continuously balanced to maintain grid frequency, voltage, and stability, avoiding sudden power losses. In vertically integrated markets, utility companies provide a bundled product of energy, reliability, and customer service, eliminating the need for explicit compensation for “ancillary services” provided by generation units to maintain system reliability.

However, with the unbundling of different segments in the electricity industry, ancillary services must be compensated to avoid compromising system reliability or power quality. For instance, with respect to reserve capacity, moving toward a liberalized market typically results in reductions to reserve capacity. If this leads to an unacceptably high risk of rolling blackouts in power systems, the creation of a market to reward capacity (separately from energy) may be justified.^7^ Further, the growth of variable renewables has given rise to new flexibility services that maintain reliability. If markets or compensation mechanisms for these services are absent, the cost of maintaining reliability with an inappropriate generation mix will rise.^18^^,^^19^^,^^20^

##### Demand-side resources

Unlocking demand-side potential is a crucial source for improving the efficiency and flexibility of electricity markets. By providing consumers with the ability and motivation to respond to electricity market prices—by adjusting or rescheduling their business activities and/or utilizing demand-side technologies that save, modulate, or generate electricity—market reform efforts can significantly lower power system costs, reduce the need for new centralized capacity additions, and facilitate the integration of customer-sited renewable resources.^21^

To enable the active participation of consumers and demand-side resources in electricity markets, two fundamental conditions must be met: (1) Provision of actionable information: Consumers need real-time information on when and how to adjust their electricity usage, which necessitates the adoption of smart meters; (2) Economic incentives: Pricing mechanisms must be established to align consumer behavior with system needs by providing financial rewards for responsive actions, such as compensation for reducing peak demand.^22^^,^^23^^,^^24^^,^^25^

#### Mechanisms to ensure long-term resource adequacy and facilitate an energy transition

Ensuring long-term resource adequacy is critical for maintaining a reliable grid and stable electricity markets. Short-term markets often operate with price caps, which limit revenues during periods of electricity scarcity, leading to a “missing money” problem that discourages investment in generation capacity. The issue of insufficient long-term investment becomes even more pronounced with the increasing share of renewables. Traditional energy-only electricity markets were primarily designed for fossil fuel-based systems, with prices set based on system marginal costs. In contrast, renewable energy sources have much lower marginal costs, as nearly all expenses are incurred upfront as capital costs and no fuel costs are incurred for generation. Moreover, high renewable energy penetrations can drive prices very low or even negative, exacerbating the difficulty generators face in recovering costs through short-term markets.^26^

To address these challenges, two main mechanisms have been proposed to induce adequate investment in generation capacity. The first is fixed-price forward contracts, whereby regulators require retailers to procure a certain percentage of their forecasted demand at different time intervals ahead of delivery through long-term contracts. These contracts provide generators with revenue certainty, hedge against price volatility, and facilitate financing for new generation to meet future demand. The second mechanism is capacity payments: generators receive fixed annual payments based on their available capacity, thereby incentivizing investment and covering fixed costs. This incentivizes long-term resource adequacy by providing financial support beyond energy market revenues.^27^

#### Regulation to address market failures and safeguard equity

The restructuring and liberalization of the electricity sector do not equate to deregulation. The California electricity crisis and subsequent power shortages in other markets have raised concerns that, without careful design and regulation, liberalized electricity markets may be unsustainable.^16^ Competitive segments must be carefully monitored to prevent the abuse of market power, especially during transitional reform phases. At the same time, the transmission segment, as a natural monopoly, continues to require government-regulated pricing. Additionally, regulatory oversight is essential to address energy equity concerns, ensuring access and affordability for vulnerable populations.

##### Mitigating local market power

The need for real-time system balancing and the impracticality of storing large amounts of electricity make electricity markets vulnerable to the exercise of market power, even by firms with relatively small market shares.^16^ Additionally, both wholesale and retail markets, as products of existing monopolies, exhibit natural tendencies toward re-consolidation. Therefore, restructuring is not and cannot be synonymous with deregulation, as markets continue to require regulatory oversight.^9^ In short, without appropriate regulation, electricity prices could escalate sharply, undermining the goals of market restructuring.

Market power issues are typically addressed through measures such as price caps and requiring a certain proportion of transactions to be handled by fixed-price long-term contracts. For regulators, determining when and whether market outcomes cause sufficient harm to certain participants is needed to justify explicit regulatory intervention. If market outcomes become excessively harmful, regulators must have the authority to temporarily suspend market operations. This requires significant subjective judgment on the part of regulators and regulatory agencies, who may lack the experience and flexibility to effectively address the wide range of potential issues, particularly during the early years of market reforms.

##### Regulating the natural monopoly in transmission and distribution

The T&D segment is the typical natural monopoly within the electricity sector. True competition would require companies to replicate each other’s wired networks, which would be extraordinarily expensive and therefore inefficient. Therefore, the regulation of the T&D segment is essential, particularly in setting T&D tariffs.^9^ Tariffs for T&D companies, as natural monopolies, are set by regulators based on underlying costs. But tariffs must also be set to encourage efficient investment in and use of the network, while addressing any revenue shortfalls.^7^ Consequently, network utility companies must, at least at times, set prices above efficient levels (such as those indicated by locational marginal price, LMP) to recover fixed costs. According to this principle, any T&D revenue gaps should be recovered from consumers rather than deducted from generators’ net income. This ensures that producers face efficient wholesale market prices, guiding their decisions regarding location, scale, and type of generation.^20^

Another critical regulatory task is ensuring equal access to transmission services for third parties. Regulated access to scarce transmission capacity and the efficient allocation of this resource are vital because transmission system investments are significant and often face opposition from communities that do not directly benefit, complicating new transmission development. There are two main approaches to addressing these issues: (1) Regulatory pathway: Imposing rules on vertically integrated utility companies to facilitate third-party access to their networks; (2) Institutional pathway: Encouraging the creation of Independent System Operators (ISOs) or Regional Transmission Organizations (RTOs). The operational independence of ISOs and RTOs from generators and retailers is crucial, as dispatch decisions directly affect the revenues of individual generators. Dispatch must align with the overall system’s best interests rather than the narrow interests of a single entity or group.^28^

##### Balancing equity and efficiency

Balancing efficiency and equity is essential for market reforms, as electricity is both a commodity and a fundamental public service. Some studies point out that the pursuit of efficiency in market restructuring could come at the expense of equity, leaving low-income populations struggling to access affordable electricity absent appropriate regulatory oversight.^11^^,^^28^ In some cases, private sector entities may lack the incentive to extend services to “unprofitable” or low-usage consumers, highlighting the need for appropriate government intervention. To promote equity, carefully designed subsidies may be employed to ensure that the benefits of market reforms reach all social groups. These mechanisms commonly include government subsidies for specific user groups, cross-subsidies from I&C users to residential users, and even cross-subsidies within residential user groups. This is especially true in developing nations, where policy interventions are vital to mitigating inequality and securing access to basic electricity services for vulnerable populations.^29^^,^^30^

### Progress of China’s electricity reform

China’s electricity sector has long operated as a government-planned system. It was vertically integrated and essentially under governmental control until 2002, when the first attempt at market-oriented reform was initiated. The 2002 reform restructured the sector by separating generation from the grid and sought to improve efficiency by encouraging competition among electricity generators.^31^ However, the reforms fell short of establishing market-based mechanisms, leaving the sector still largely governed by planning. New capacity investment needed approval from the central government, electricity production was allocated by the local government, and prices were predetermined by provincial governments.

**Figure 1:**
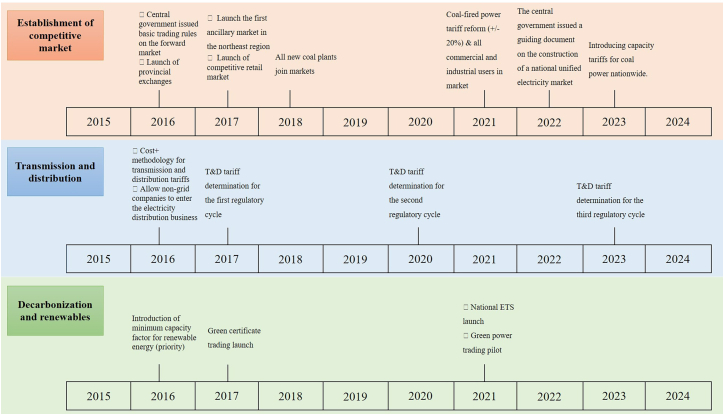
China’s power sector reform milestones since 2015

In 2015, China inaugurated a new round of reform, which broadly focused on fostering price competition in generation and retail, enhancing the regulation of grid costs and services, expanding inter-regional transmission, and promoting renewable electricity. The reform so far has achieved several impressive outcomes. The role of the market in resource allocation has been significantly enhanced, wholesale and retail markets have been established, the T&D pricing system has been refined, and renewable energy has gradually entered the market. China’s reform process illustrates a tailored approach to market rule design, reflecting unique national and regional circumstances. While some features align with international practices, significant distinctions remain, such as ongoing state involvement, limited trading activities, an emphasis on long-term contracts over spot markets, high market concentration, and a preponderance of state-owned entities.^32^^,^^33^ China’s electricity sector is currently entering a transitional phase where production quotas coexist with competitive market elements. Key milestones since the 2015 reform are depicted in Figure 1.

#### Wholesale market reform

**Figure 2:**
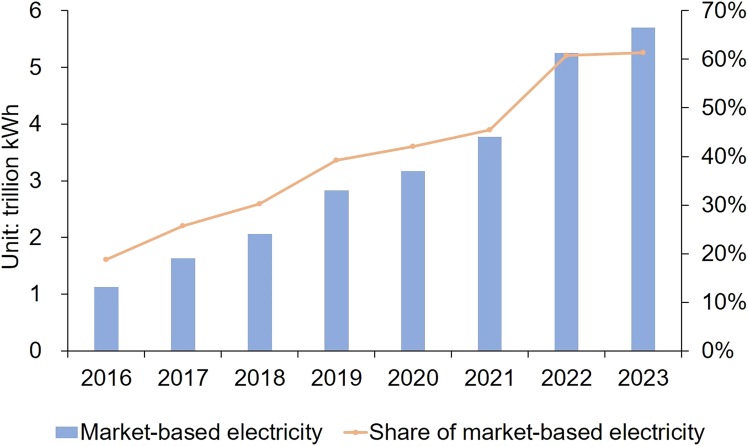
Market-based electricity and share of market-based electricity *Source*: China Electricity Council.

The aspect of reform receiving the most attention was the establishment of wholesale electricity markets. Prior to these reforms, while the sector had undergone vertical unbundling and generation was divided horizontally among multiple producers, the system operated under a “single buyer” model. In this setup, vertically integrated T&D companies monopolized both the procurement of energy from generators and the supply to end users. Prices and generation volumes were strictly regulated by the government. Generator dispatch followed general principles of maintaining “equal shares” across generators and ensuring revenue sufficiency.^34^ Specifically, local governments forecasted electricity consumption for the next year and allocated a total generation amount to each power plant based on an approximately equal quota. Under this “fair dispatch” rule, thermal generating units of a similar type and capacity were assigned an equal amount of annual operating hours, to ensure the equity of power generation.^35^^,^^36^ Provincial “benchmark tariffs” were fixed by the National Development and Reform Commission (NDRC) based on expected returns, inflation, and other socio-economic concerns.^36^ Since 2015, a diversified market structure has emerged, encompassing long-term, spot, and ancillary service markets. By the end of 2021, China’s central government required all coal-fired generation to be traded through market-based mechanisms. Accordingly, market-based electricity transactions increased from 13% of total consumption in 2015 to 61% in 2023 (Figure 2).

China’s wholesale market reform can be described as an experimental governance process, where the central government provides a broad yet rudimentary framework while actively encouraging the implementation of pilot electricity markets at the local level. Consequently, the development of wholesale electricity markets varies significantly across provinces (Table 1). Even so, a typical reform path adopted by many provinces is as follows: (1) build a forward (physical) market, expand market access, increase the amount of market-based electricity trading, and cultivate participants’ market awareness; (2) build an ancillary service market (for frequency regulation, energy reserve, and others) and perhaps a peak regulation market, depending on the extent of renewable curtailment; (3) build a spot energy market closely integrated with the forward market, which could involve a mix of financial and physical contracts; (4) build a capacity market and financial forward markets involving futures and options,^7^^,^^37^^,^^38^^,^^39^ but so far, no provinces has achieved this step, and all provinces currently implement administrative capacity compensation mechanisms.

**Table 1:** Pathways in China’s electricity wholesale sector

	Market type	Province
Stage 1	Medium- and long-term market	All except Tibet
Stage 2	Ancillary services market	All except Tibet
Stage 3	Spot market (official operation)	Shanxi, Guangdong, Gansu, Shandong, western Inner Mongolia
Stage 4	Capacity market (capacity compensation mechanism currently)	Initially, Shandong and Guangdong; in 2024, all except Tibet

##### Medium- and long-term market

China first built a medium- and long-term market rather than a spot market at the start of the 2015 reform. Spot markets for electricity are central to the development of wholesale markets, as they provide price signals for efficient dispatch and the efficient operation of medium- and long-term markets.^7^ However, price volatility in spot markets is high, and the Chinese government, wary of volatile electricity prices or even electricity shortages, began the reform by establishing the medium- and long-term market. Most contracts in China’s medium- and long-term market are on monthly or longer horizons and are physical contracts for energy specifying only a price and total amount, which must later be decomposed into power curves and implemented into actual power system dispatch.^38^ Since the 2015 reform, provinces have explored medium- and long-term electricity trading, with most provinces specifying that such trading must account for 90–95% of market-traded electricity.^40^ China’s central government issued basic trading rules for the medium- and long-term market at the end of 2016, regulating trading behaviors across provincial markets nationwide and further promoting market development.^41^ Except for Tibet, all provinces have established a medium- and long-term market. Notably, China has set an upper and lower price limit for its medium- and long-term electricity markets, with fluctuations allowed to range from 10 to 20% of the current benchmark price in each province.

##### Ancillary services market

Ancillary services refer to services beyond electricity production and transmission that ensure the safe and stable operation of the power system. These services include primary frequency regulation, reactive power regulation, reserves, and ramping, which are closely tied to real-time power quality maintenance. Currently, the peak-shaving market is a unique ancillary service market in China, and its most critical ancillary service product. Established on a transitional basis to address real-time power balance issues during the early stages of spot market development, the peak-shaving market will be phased out when the spot market becomes fully operational.

**Figure 3:**
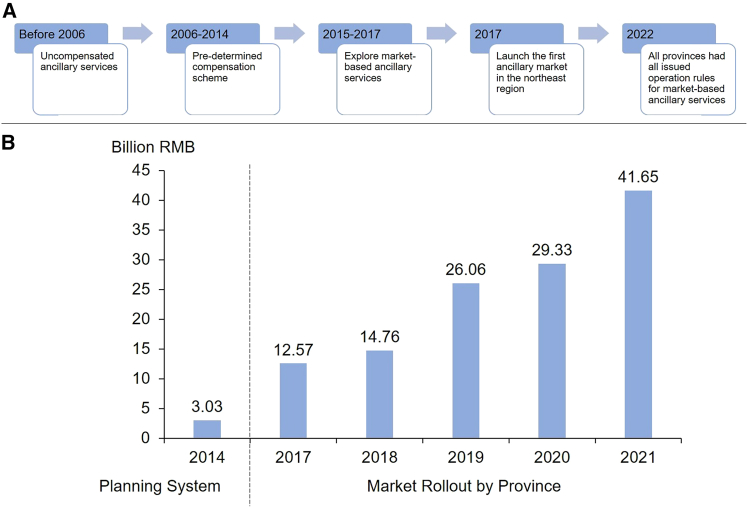
Evolution of the ancillary services market reform in China (A) China’s ancillary service market development. *Source*: National Energy Administration. (B) China’s ancillary service costs by year.^44^

Figure 3A illustrates the evolution of ancillary services in China. Before the first round of electricity sector reform in 2002, these services were provided free of charge by generation units. At that time, almost all generation units were owned by the State Power Corporation, eliminating the need for specific units to be compensated. After the electricity sector restructuring, generation units became affiliated with independent power companies with individual profit objectives. Since 2006, ancillary service providers have received compensation, albeit at a low rate. Generating companies providing these services were reimbursed under the principle of “cost compensation plus reasonable returns,” with costs distributed among units that do not participate in ancillary service provision.^42^^,^^43^

As China’s share of renewable energy increased, demand for ancillary services rose. Low, non-market-based compensation rates failed to incentivize flexible generation units to provide sufficient ancillary services, creating an urgent need for market-based price signals. Such signals must reflect both the grid value of ancillary services and the rising costs of provision to encourage retrofitting for flexibility among generators and to bridge service gaps. Against this backdrop, the ancillary services market was proposed in 2014 and became operational in northeast China (including Heilongjiang, Jilin, and Liaoning) in 2015, which primarily focused on peak-shaving service. It has since expanded to all provinces.

The ancillary services market typically operates in three steps: (1) Demand declaration: grid companies announce the demand for ancillary services. (2) Bidding process: market participants submit prices and capacities for the services they can provide, considering the opportunity cost between ancillary services and the spot market (as capacity allocated to ancillary services forgoes revenue opportunities in the spot market). In the early stages, only thermal and hydropower units could participate. By 2024, energy storage and virtual power plants were added as market participants, adding a new potential revenue stream for these resources.^45^ (3) Sorting and clearing: bids are ranked from lowest to highest price and cleared at the marginal price.

The allocation of ancillary service costs is a key challenge in developing this market. From a theoretical perspective, there are three cost-sharing models: (1) All costs borne by generators: this distorts the energy market, as generators must consider ancillary service costs when bidding in the spot market, thereby preventing marginal-cost bidding and reducing market efficiency. (2) Costs shared by generators and users: while still causing some distortion, this approach is more acceptable. (3) All costs borne by users: the ideal model, involving no market distortion, but may face initial consumer resistance due to electricity bill increases. In practice, regardless of who initially bears the ancillary service costs, these are eventually passed on to users, as generators adjust their bids in other markets to recover these costs.

The general compensation principle is “providers benefit,” and the cost-sharing principle is “beneficiaries bear costs.” However, fully implementing these principles remains difficult. Before 2009, ancillary service costs were borne primarily by coal-fired and hydropower units. After 2009, wind and photovoltaic units were gradually included. Since 2014, power plants exporting electricity to other provinces have begun sharing ancillary service responsibilities based on exported electricity. Since 2022, efforts have been made to include market-based consumers in cost-sharing, excluding residential, agricultural, institutional, and essential public service consumers. In 2024, new participants—such as energy storage plants and virtual power plants, which integrated generation and consumption—began sharing costs based on their electricity input (generation side) or output (user side). As shown in Figure 3B, ancillary service costs have increased more than 10-fold since the market was established, rising from 3 billion yuan in 2014 to 41.6 billion yuan in 2021, accounting for approximately 1.5% of China’s electricity costs.

Cost-sharing mechanisms for ancillary services vary based on the status of provincial spot markets. In provinces without continuously operating spot markets, all costs are borne by generators, while in provinces with continuously operating spot markets (i.e., Guangdong, Shanxi, Shandong, Gansu, Fujian, Western Inner Mongolia, Zhejiang, Hubei), costs are shared between generators and users.^45^ Typically, costs are divided equally (1:1) between generators and consumers, and then distributed proportionally within each group based on electricity production or consumption. Why does this cost-sharing mechanism differ between provinces with and without spot markets? A plausible explanation is that consumers in spot markets play a more active role in optimizing the power system compared to their counterparts in non-spot market regions. Real-time price signals in spot markets offer users both the flexibility and financial incentives to adjust their electricity consumption. Consequently, it makes economic sense that users in spot market provinces would take on ancillary service cost-sharing responsibilities ahead of users in non-spot market provinces.

##### Spot market

The core of electricity market development lies in the spot market. Before the operation of its spot market, China introduced a peak-shaving market as a partial substitute to incentivize generating companies to adjust resources and provide sufficient flexibility for the system, ensuring real-time supply-demand balance. The peak-shaving and spot markets differ in significant ways, with the pricing mechanism being the fundamental distinction. In the peak-shaving market, ancillary services are typically bid unilaterally by providers in the day-ahead or intra-day time frame. The dispatching authority then ranks bids from low to high according to predetermined rules and utilizes the required capacity. As a result, market-based ancillary service prices are determined through competitive allocation driven solely by supply-side bids, with no participation from the demand side in pricing. In contrast, the spot market adopts a more integrated approach, enabling both buyers and sellers to participate actively in pricing through price and quantity bids. This dynamic interaction between supply and demand enhances market efficiency and transparency.

China’s spot market started as a pilot in a few provinces. In 2017, the central government selected eight regions—Guangdong, western Inner Mongolia, Zhejiang, Shanxi, Shandong, Fujian, Sichuan, and Gansu—for the first batch of electricity spot market pilots. In 2021, spot market pilots expanded to Shanghai, Jiangsu, Anhui, Liaoning, Henan, and Hubei. In 2023, based on operational experience from pilot provinces, the central government issued basic rules of electricity spot market operation for the first time. As of year-end 2024, China’s electricity spot market has achieved near-complete provincial coverage, with markets in Shanxi, Shandong, Gansu, and Guangdong now officially in operation. Table 2 provides an overview of the status of each market in 2024. In provinces where the spot market is not yet fully operational, a trial spot market may coexist with a peak-shaving market. Provinces such as Sichuan and Henan typically require thermal power units to bid 50% of their capacity into the spot market, with the remainder submitted to the peak-shaving market.^46^^,^^47^ The purpose is to provide stable compensation, allowing owners of thermal generation to gradually adapt to reforms and reducing their resistance to the changes.

**Table 2:** Status of China’s spot power markets

Status	Province
Official operation	Shanxi, Guangdong, Gansu, Shandong
Continuous trial operation	Fujian, Western Inner Mongolia, Zhejiang, Hubei
Long-term trial operation (more than one month)	Sichuan, Jiangsu, Anhui, Liaoning, Henan, Shaanxi, Hebei
Short-term trial operation (less than one month)	Southern region (Guangdong, Guangxi, Yunnan, Guizhou, Hainan), Ningxia, Chongqing, Hunan, Jiangxi
Simulation test	Jilin, Tianjin, Qinghai, Heilongjiang, Xinjiang, Shanghai
No plan	Beijing, Tibet, Eastern Inner Mongolia

*Source*: IEA^48^; Provincial Development and Reform Commissions.

On the supply side, thermal power generators primarily participate in spot market transactions by bidding on both volume and price. Notably, many regions have opened their electricity spot markets to a broad range of participants. Entities such as centralized renewable energy facilities, independent energy storage systems, virtual power plants, pumped storage, and nuclear power plants have gradually joined the spot market, increasingly engaging in spot trading through volume-and-price bidding mechanisms. On the demand side, while Gansu has adopted volume-and-price bidding, volume-only bidding continues to dominate. Nonetheless, provinces such as Guangdong and Shandong are actively experimenting with volume-and-price bidding models. Time-based price signals in the spot market are now steering users away from traditional “demand-driven electricity usage” toward “price-sensitive electricity usage,” enhancing resource allocation efficiency. For example, in Gansu, the daily peak load has shifted from the evening to midday (when photovoltaic generation surges), enabling “peak shaving” and “valley filling” of 2.4 GW (GW), equivalent to 10% of its total load.^49^

##### Capacity compensation mechanism

Due to the intermittent and volatile characteristics of renewable power generation, it is necessary for regulating units, primarily coal-fired power plants and energy storage units, to manage fluctuations and ensure sufficient power supply when the output of wind and photovoltaic units declines. However, as the proportion of renewable energy in the power mix increases, the profitability of coal-fired regulating units diminishes under traditional electricity pricing mechanisms, making it difficult for them to sustain normal operations, let alone make long-term investments to ensure future capacity adequacy. The main reasons are 2-fold: first, renewable energy has near-zero marginal costs, which significantly depresses spot market electricity prices when introduced at scale; and second, large-scale renewable energy generation crowds out the economic dispatch space for other power sources. Additionally, due to price caps, generating units cannot earn substantial scarcity rents during periods of tight supply. To address this issue and support the flexible operations needed to integrate renewables, China has introduced a capacity compensation mechanism aimed at enabling coal-fired units to recover stranded costs and maintain stable operations.^50^

In 2020, Shandong and Guangdong were the first provinces to trial capacity compensation for coal power units. By 2024, this mechanism had expanded nationwide, transitioning coal power pricing from a single-part tariff to a two-part tariff, with the capacity component set by the government at a rate of 100–165 yuan/kW annually. In seven provinces where coal power plants face greater financial losses, such as Yunnan and Sichuan, the proportion of fixed coal power costs recovered through capacity pricing is set at 50%, while in other regions it is set at 30%. According to Geng et al.,^51^ current compensation standards can ensure cost recovery for coal-fired power units. However, the current mechanism does not differentiate between units that have already recovered their costs and those that have not, applying a uniform standard that may potentially reduce the effectiveness of targeted financial support. Moreover, the current mechanism solely targets coal-fired power enterprises. In practice, other adjustable power units could also be compensated, especially energy storage. New energy storage systems are characterized by flexible operation and strong regulation capabilities, but in the absence of a well-developed market mechanism, policy support is needed to encourage their development.^52^^,^^53^

#### Reform on the consumer side

**Figure 4:**
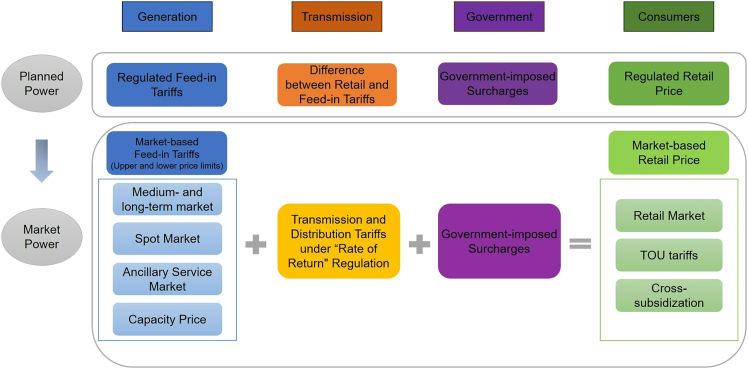
China’s electricity pricing mechanism China’s electricity pricing system is currently in transition from a centrally planned framework to a market-based mechanism, and at this stage, a degree of dual-track pricing still exists. *Note*: Government-imposed Surcharges refer to state-mandated fees levied through electricity tariffs to finance specific public initiatives or policy objectives. These charges are collected by grid operators on behalf of the government and ultimately borne by end-consumers via their electricity bills, covering areas such as water conservancy, resettlement, renewable energy, and rural grid upgrades.

Historically, China’s electricity sector has operated under a “single buyer” model, where grid companies purchase and resell all electricity (with government involvement through quota- and price-setting). The 2015 reform marked a significant shift on the consumption side, notably by easing the monopoly franchise rights previously held by utilities. By the end of 2021, the central government required, in principle, that all I&C consumers participate in market-based electricity trading. Today, grid companies no longer serve as the sole purchasing channel for consumers. I&C users can now access electricity through two primary pathways: direct transactions with generators via wholesale markets or indirect participation through long- or short-term contracts with retailers.^54^ Non-market consumers—primarily households, agriculture, and public service sectors—continue to rely on grid companies, purchasing electricity at regulated tariffs. In 2022, I&C consumers purchased about 4.8 billion MWh of electricity through the market, accounting for about 90% of their electricity consumption. With ongoing market development, innovative demand-side mechanisms such as virtual power plants have emerged, further enhancing demand-side efficiency and flexibility. Figure 4 shows China’s electricity pricing mechanism.

##### Retail market

Since the 2015 electricity reform, consumers have gained increasing access to the market through retail companies. The retail market aims to diminish the privileged position of monopoly suppliers by providing direct consumer access, fostering competition among retail suppliers for customer contracts. Consumers now have the option to participate in wholesale markets either directly or indirectly by signing long- or short-term contracts with retailers. The expansion of market-based electricity trading has driven growth in the number and activity of retail companies. By the end of 2022, there were 15,000 registered electricity retailers. In 2018 alone, retail companies facilitated transactions exceeding 200 million MWh, accounting for nearly 10% of electricity transactions. Ideally, retail electricity tariffs would be tied to wholesale market prices, particularly spot market prices, to encourage efficient consumption and provide accurate locational signals.^37^ In practice, however, retail prices often deviate from this principle. Retail contracts rarely link directly to wholesale price fluctuations; instead, retailers and customers usually negotiate fixed tariffs for the upcoming year, which remain unchanged regardless of subsequent variations in wholesale prices. Retailers generate most of their income by capitalizing on this price differential, making it their primary source of revenue rather than relying on service fees.

##### Demand-side management

China began implementing demand-side management relatively early in its reform efforts, but progress has been slow. China’s time-of-use (TOU) pricing policy dates to 2003, aiming to guide consumers to use more electricity during off-peak periods and less during peak periods. Following recent reforms, demand-side management has seen widespread adoption. By 2021, 29 out of 33 provincial grids had implemented TOU pricing. Initially, TOU pricing only differentiated between peak and off-peak periods; gradually it became more detailed, distinguishing among peak, shoulder, and off-peak periods. The price gap between peak and off-peak periods has been widening. For example, in Guangdong, the peak/shoulder/off-peak (PSO) price ratio expanded from 1.5:1:0.5 in 2022 to 1.7:1:0.38 the next year.^55^ The contracts will specify the price ratios that consumers must pay. Currently, TOU tariffs are predominantly applied to I&C sectors. One reason is that residential consumers are relatively insensitive to time-varying tariffs.^56^ In contrast, I&C customers are typically more responsive to price changes.^57^^,^^58^ Another reason is the relatively low share of electricity consumption by residential and agricultural sectors.

In 2012, Shanghai, Suzhou, Beijing, Tangshan, and Foshan were the first cities approved for demand-side management pilots and gradually explored market-based demand response. Market rules explicitly stipulated that demand response participants would be rewarded for reducing loads during peak periods.^59^ However, demand response, and especially virtual power plants, remain in their infancy. In summary, demand-side management and electricity market operations are currently not well integrated, and demand-side management remains largely reliant on administrative TOU pricing.

#### Reform in transmission and distribution

Reforms in the T&D segment have been notably more successful. A key objective has been to redefine the role of grid companies, which no longer function as single buyers in the wholesale market or single sellers in the retail market. Prior to these changes, T&D tariffs were embedded within retail electricity prices. The new reforms have established standalone T&D tariffs for intra-provincial, inter-provincial, and inter-regional transactions, transforming these tariffs into the grid companies’ primary revenue source and replacing the previous model of charging customers based on the buy-sell price differential. The tariff structure is regulated using a rate-of-return framework, aimed at curbing the profitability of grid companies. T&D tariffs in China are typically reviewed and adjusted every three years. During these intervals, grid companies can achieve higher-than-allowed returns if they successfully lower costs, creating a built-in incentive for efficiency improvements. Three rounds of tariff regulation have been conducted since 2015. The first round, conducted from 2016 to 2018, reduced approximately 128.4 billion yuan of unrelated and unreasonable costs, accounting for about 15% of the original T&D expenses. The average T&D price was lowered by 0.014 yuan/kWh compared to the previous price differential between feed-in tariff and retail tariff.^60^

New reforms support private investment in electricity distribution, although the T&D system is still operated mainly by grid companies. The Incremental Power Distribution Reform Pilot has been launched since 2016, aiming to introduce competition to the distribution segment. By the end of 2022, the National Energy Administration had issued distribution business licenses to 237 owners of private distribution projects.^61^ Nonetheless, the actual impact of Incremental Power Distribution Reform has been constrained. This is largely attributable to the entrenched dominance of the grid companies, which continue to hold a superior market position relative to the incremental operators. Incremental distribution entities generally lack independent operational capacity and remain highly dependent on incumbent grid companies for core functions such as transmission, dispatching, trading, and settlement. In some cases, grid companies have imposed implicit barriers, limiting the autonomy of incremental distributors.

#### Reform in market integration

The electricity sector in China has historically been fragmented, primarily due to two key factors. First, local protectionism has led provincial governments to prioritize local power generation while limiting competition from other regions to achieve economic and political objectives. Second, significant disparities in market rules and the pace of reforms across provinces have further deepened fragmentation, as reforms are often piloted in specific regions before being implemented nationally. China’s energy transition has been likened to an iterative process of negotiation between central and provincial actors, involving complex, non-zero-sum dynamics. The spatial politics and accountability structures in China have played a critical role in guiding electricity market reforms. In the country’s decentralized policy framework, provincial actors hold substantial influence.^62^ While the central government provides overarching guidance, the specifics of reform initiatives are largely left to provincial authorities, leading to divergent reform trajectories across regions.^7^^,^^63^ The 2015 reform aimed to address this fragmentation by fostering market integration. Given the significant disparities in energy resources and economic development across regions, electricity supply, and demand are unevenly distributed across China. A unified national market is therefore essential to optimizing electricity resource allocation on a broader scale.

In early 2022, China’s central government issued a guidance document on the construction of a unified national electricity market, proposing its near-completion by 2030.^64^ Two power exchange centers have been established to support regional power markets in the State Grid and South Grid regions. Attempts at market integration are occurring in China’s medium- and long-term, spot, and ancillary services markets. In the long-term market, inter-provincial trading has been running continuously since March 2022, with the total trading volume reaching 1.04 billion MWh for the year.^65^ In the spot market, the Southern market was the first to begin trial operations in 2018, and entered the monthly trial operation phase in November 2024.^66^ Additionally, the State Grid’s inter-provincial spot market launched its pilot phase in January 2022, achieving an annual trading volume of 27.8 million MWh and involving over 6,000 generators across 21 regions and buyers from 25 provinces. In the ancillary service market, pilot operations for reserve and peak-shaving markets in the Southern region began in 2021.^67^ In 2022, Sichuan and Chongqing merged their markets, enabling power plants to provide ancillary services across provincial boundaries.

#### Reform for integrating renewables

**Figure 5:**
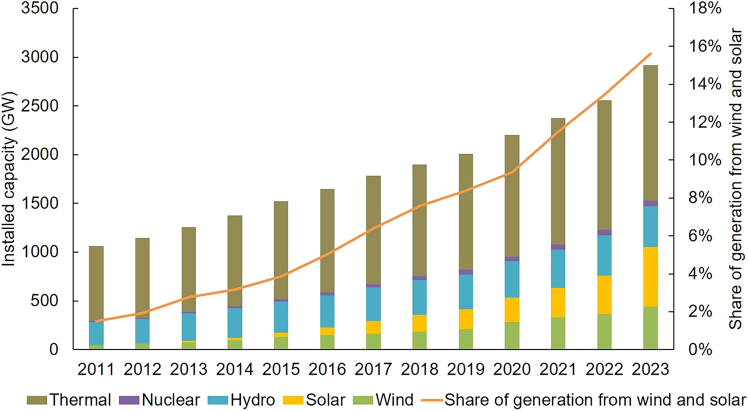
China’s installed generation capacity by type *Source*: National Bureau of Statistics.

Transitioning to low-carbon resources is both a goal and guiding principle for the future development of China’s electricity sector. As of 2023, China leads the world in renewable energy capacity, with wind and solar installations reaching 441 GW and 609 GW, respectively. In 2023, China’s market-based trading of renewable electricity reached 684.5 billion kWh, representing 47.3% of total renewable power generation.^68^ Figure 5 illustrates the rapid growth in China’s renewable energy installations. Historically, the country’s low-carbon transition has been driven by fixed feed-in tariffs and guaranteed purchase policies, with minimal integration into competitive electricity markets.

As wind and solar continue to account for a growing share of generation, China is increasingly embracing market-based mechanisms for renewable energy trading. Beyond integrating renewables into long-term and spot markets, specialized “green electricity” trading platforms have been established, enabling environmentally conscious users to negotiate prices directly with generators, although trading volumes remain small. In the State Grid regions, the first year of green electricity trading (up to September 2022) resulted in cumulative transactions of 17.6 million MWh, with environmental value reflected in a 0.03-0.05yuan/kWh premium over local coal-fired benchmark tariffs.^69^ Additionally, China employs a distinctive form of generation rights trading, where renewable or cleaner generators replace quotas allocated to inefficient and polluting units.^70^^,^^71^ This unique mechanism differs from other contract substitution models seen in countries such as the United States, Australia, and the UK. In 2025, to encourage the full market participation of renewable energy and mitigate revenue volatility associated with its intermittency, China implemented a contract-for-difference (CFD) scheme, mandating that all wind and solar generation be traded through market-based mechanisms.

### Electricity market performance since the 2015 reforms

#### Enhancing efficiency in power generation

The 2015 market-oriented reform has widely been acknowledged to have improved operational efficiency on the generation side. Most studies simulate counterfactual scenarios post-reform, analyzing changes in coal consumption and production costs to evaluate efficiency gains. These studies estimate that the shift from fairness-based dispatch to economic dispatch has resulted in a 3–15% improvement in production efficiency.^4^^,^^72^^,^^73^^,^^74^^,^^75^^,^^76^^,^^77^ Dispatch reform represents a step toward market allocation, with value prioritized based on energy efficiency, incentivizing reduced fuel consumption and lower marginal costs. A smaller number of studies employ empirical analyses using power plant operational data from specific regions or provincial panel data before and after the reform. These analyses confirm that China’s market reforms have enhanced efficiency in the power sector.^78^^,^^79^^,^^80^

##### Smaller-capacity units see greater efficiency gains

**Figure 6:**
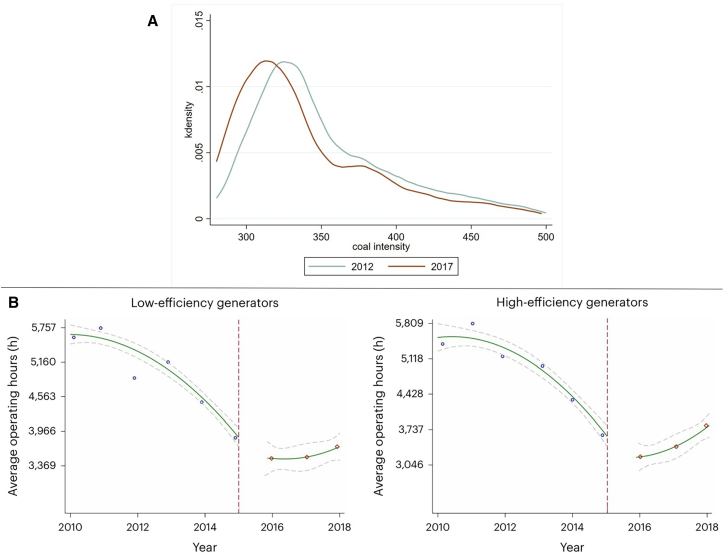
Efficiency improvement in power generation since China’s 2015 electricity reform (A) Coal intensity distribution in power plants. Coal intensity represents the standard coal consumption per power generation (g/kWh). (B) Changes in average operating hours of low-efficiency and high-efficiency generators in Guangdong before and after reform. *Source*: Xiang et al.^79^

We use national power plant data to further validate these efficiency improvements. Figure 6A shows the distribution of coal intensity (coal used per kilowatt of electricity) for all of China’s coal-fired plants in 2012 and 2017. It can be seen that the distribution of coal intensity of power plants has clearly shifted to the left.

To further test this relationship between coal intensity, reform, and installed capacity, we estimate the regression on plant-level data for all coal power plants in the country from 2012 to 2017.

The results of the regression are reported in Table 3. The first column of Table 3 suggests that the average coal intensity of coal-fired plants decreased by 4% after the reform. Columns 2 and 3 introduce the interaction term (Reform × Capacity) to investigate whether unit capacity influences the reduction in coal intensity during the reform period. The results show that the positive coefficient of the interaction term is significant (at the 1% level) whether in a one-way fixed-effects model or in a two-way fixed-effects model. This implies that the larger the unit capacity, the less significant the reform’s impact on reducing coal intensity, and that reform has mainly driven efficiency improvements in smaller-capacity units. This could be attributed to the substantial potential for efficiency enhancement in smaller-capacity units, which typically have higher marginal costs than larger units and must reduce fuel costs to compete more effectively in electricity markets. In the short term, market-oriented reforms have indeed pressured small-capacity generating units to improve their efficiency. However, over the long run, their inherent disadvantage in marginal costs cannot be fully overcome through efficiency gains alone, making them vulnerable to being phased out of the market. Looking ahead, the long-term survival of such units may depend on their ability to adapt—either by undergoing flexibility retrofits to meet the operational challenges posed by high shares of renewables, or by transitioning to combined heat and power (CHP) or industrial steam supply to offset the limited profitability of electricity generation alone.

**Table 3:** Regression results of coal intensity and reform

Regression	(1)	(2)	(3)
Reform	−0.0405∗∗∗ (0.00265)	−0.0715∗∗∗ (0.0135)	−0.0896∗∗∗ (0.0135)
Capacity	−0.00922∗ (0.00477)	−0.0137∗∗∗ (0.00487)	−0.0121∗∗ (0.00482)
Reform∗ Capacity	–	0.00389∗∗∗ (0.00107)	0.00393∗∗∗ (0.00106)
Constant	5.969∗∗∗ (0.0585)	6.010∗∗∗ (0.0598)	6.005∗∗∗ (0.0592)
Province fixed effect	YES	YES	YES
Year fixed effect	YES	NO	YES
Observations	6,074	6,074	6,074

Standard errors in parentheses. ∗∗∗*p* < 0.01, ∗∗*p* < 0.05, ∗*p* < 0.1. *p* values are for a two-sided test based on normal distribution. “Yes” denotes that the fixed effect is controlled in the model; “No” is vice-versa.

##### Efficiency improvements are largely attributed to dispatch reforms prioritizing high-efficiency units

Several studies have analyzed the reasons for the increased efficiency of power generation. The main reason for this increase is the shift from “equal share” dispatch to market-based dispatch, which increases the operating hours of efficient generators.^81^^,^^82^ Equal share dispatch has often been criticized for its inefficient use of polluting power plants.^7^^,^^83^ Market-based dispatch encourages more efficient generators with lower fuel costs to produce more electricity.^84^^,^^85^^,^^86^^,^^87^ As shown in Figure 6B, in Guangdong, the operating hours of high-efficiency power units have risen significantly since the 2015 reforms.^79^ Cui et al.^82^ construct a counterfactual market reform scenario based on micro-panel data of coal-fired units in five of China’s southern provinces, finding that coal-fired power generation costs and carbon emissions both decrease by about 2.4% compared with the 2015 baseline scenario. Using the log mean divisia index (LMDI) decomposition methodology,^88^ they demonstrate that these savings are mostly due to improvements in allocation efficiency (about 45%), followed closely by fuel efficiency improvements (about 40%) and reductions in the self-consumption rate (about 14%).^82^

##### Potential for efficiency gains is constrained by incomplete marketization

China’s transition to market-based dispatch remains incomplete, and while efficiency has improved it has not been fully realized.^80^ The power system currently operates under a “semi-planned and semi-market” dispatch approach in China, where “in-plan” generation is pre-allocated to generators by local governments and “out-of-plan” generation is determined through market competition, a dynamic that limits the potential of marketization. In addition, market reforms have faced certain political challenges, with local governments favoring local enterprises over central state-owned enterprises in their allocated generation quotas. This protects small coal-fired and natural gas generators, lessening their motivation to improve generation efficiency even after reforms. Using generator-level data of China’s Southern Power Grid region in 2019, Xiang et al. (2023)^89^ found that only half of the potential CO_2_ emission reductions and social welfare gains have been realized due to politically allocated generation dispatch.

##### Transmission and distribution efficiency have not significantly improved

Beyond the generation sector, several studies have examined the impact of the 2015 reforms on T&D efficiency. Zheng et al. (2021)^78^ found that the impact of reforms on T&D efficiency was not statistically significant based on the line loss rate. One possible explanation for this is that China’s ongoing effort to upgrade its T&D infrastructure is largely independent of the 2015 reforms. Another possible explanation is that China’s T&D system was already relatively efficient, leaving limited scope for further improvement.^90^

#### Incomplete marketization limits the effectiveness of electricity pricing reforms

The 2015 reform sought to transition pricing from administrative control to market-driven mechanisms. However, China’s pricing system remains largely regulated, with limited sensitivity to cost fluctuations or supply-demand dynamics. While the nascent spot market effectively captures market dynamics, including supply-demand fluctuations and coal price changes, and thereby offers a glimpse into the potential of market-driven mechanisms, the small size of this market limits its effect on overall prices. I&C electricity prices have decreased significantly since 2015, but this is mainly due to government-driven administrative interventions rather than improved market efficiency. China’s T&D tariff reforms, which focus on reducing grid costs and establishing rate-of-return regulation, have significantly contributed to price reductions.^91^ Additionally, TOU pricing reforms have shown promise in improving social welfare by optimizing resource allocation and reducing capacity investment costs. Despite these advancements, the incomplete marketization of China’s electricity sector continues to constrain potential benefits from efficiency gains and cost-reflective pricing, as discussed in the following sections.

##### Electricity prices are mainly set by government regulation, with limited connection to costs or supply-demand dynamics

An important goal of the 2015 reform is to determine electricity prices through markets, not government action. Nevertheless, some studies find that China’s electricity pricing is still largely dependent on government regulation, with costs and supply-demand dynamics having little impact. Feed-in tariffs are adjusted infrequently and inadequately, leaving coal-fired power producers exposed to coal price volatility. Regulatory lag has limited the profit margins of coal-fired power enterprises.^92^^,^^93^^,^^94^ If price regulation were relaxed, Liu et al.^95^ found that, assuming other conditions remained unchanged, it would result in higher electricity prices and lower coal prices.

With fuel costs accounting for about 70% of the total cost of coal-fired power generation, the degree to which electricity prices mirror fluctuations in coal prices serves as a key indicator of the effectiveness of market reforms. To test the relationship between coal prices, reform, and end-user prices, we estimate the regression on province-level monthly changes in end-user prices and coal prices.

**Figure 7:**
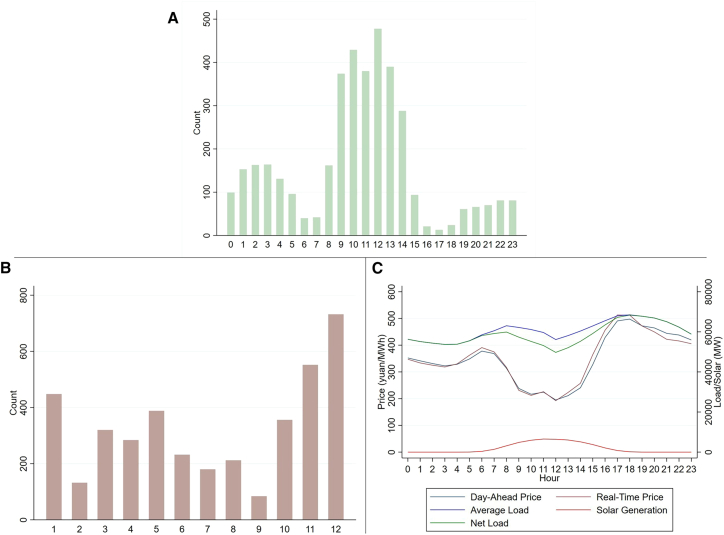
Spot market electricity prices (A) Instances of negative electricity prices in Shandong by hour (2023). (B) Instances of negative electricity prices in Shandong by month (2023). (C) Annual average load and spot prices in Shandong. The “Duck Curve” refers to the daily net load curve in power systems (total electricity load minus renewable generation), characterized by a midday dip (due to high solar generation) and a steep evening ramp-up in demand. It underscores the operational challenges posed by renewable energy intermittency.

The results are reported in Table 4. As Table 4 suggests, the reform resulted in a 0.5% reduction in end-user electricity prices from 2014 to 2020. Changes in local coal prices, however, had no statistically discernible effect on electricity prices, as shown in the second column, reflecting that electricity prices did not keep pace with underlying cost changes and remain strongly regulated by the government. The third column in Figure 7 shows that the government’s price-setting mechanism did not change after 2015. When the change in coal price interacts with the reform, the result suggests that post-reform, coal costs still do not have a statistically significant effect on electricity prices.

**Table 4:** The result of electricity price and coal price: Inadequate cost reflection in electricity pricing

Regression	(1)	(2)	(3)
Reform	−0.00464∗∗∗ (0.00144)	−0.00467∗∗∗ (0.00144)	−0.00480∗∗∗ (0.00145)
ΔCoal_price	–	0.00172 (0.00611)	0.0163 (0.0148)
Reform∗ ΔCoal_price	–	–	−0.0176 (0.0162)
Observations	2,100	2,100	2,100

Note: Standard errors in parentheses. ∗∗∗*p* < 0.01, ∗∗*p* < 0.05, ∗*p* < 0.1. *p* values are for a two-sided test based on normal distribution.

Additionally, dampened electricity price effects can be partly attributed to the fact that long-term contracts constitute the vast majority of market-based transactions. While these contracts do allow price fluctuations within a band near the government benchmark price, the upper and lower limits constrain the degree of fluctuation. Moreover, the benchmark price itself is slow to adjust to changes in coal prices. For example, Zheng et al.^78^ demonstrated that from 2003 to 2018, coal prices had no significant effect on provincial benchmark prices for thermal power. As a result, coal-fired generators cannot fully pass on fuel costs to end-users.

##### Spot market prices reflect market conditions

China’s spot market—still in its infancy, with fewer pilot provinces and a relatively small share of electricity transactions—is working reasonably well, with prices that reflect supply and demand at different moments. Taking the Shandong spot market as an example, we use data from December 2021 to April 2024 and sum 15-min interval data to daily data for the regression. Table 5 shows the regression results, indicating that spot market prices are sensitive to both coal price and demand and suggesting they accurately capture supply-demand fluctuations.

**Table 5:** Regression results of spot market price and coal price

Variables	Real-time price	Day-ahead price
Coal price	0.581∗∗∗ (0.198)	0.553∗∗∗ (0.180)
Demand	2.292∗∗∗ (0.116)	2.080∗∗∗ (0.102)
R-squared	0.400	0.421
Observations	849	853
Year FE	YES	YES
Month FE	YES	YES

Note: Standard errors in parentheses. ∗∗∗*p* < 0.01, ∗∗*p* < 0.05, ∗*p* < 0.1. *p* values are for a two-sided test based on normal distribution.

With the growing share of wind and solar power, weather fluctuations (e.g., wind speed and solar radiation) can cause sudden surges in power supply that outpace demand adjustments, leading to negative spot market prices. For example, Shandong experienced 22 continuous hours of negative prices during the May Day holiday in 2023, drawing significant attention. Negative prices typically result when supply exceeds demand during periods of low consumption. On an hourly scale, they usually occur during the daytime (Figure 7A), while seasonally, they are more common in winter (Figure 7B). The occurrence of negative prices demonstrates the spot market’s ability to reflect real-time supply conditions. However, given China’s relatively low share of wind and solar power generation, the frequent appearance of negative prices reveals a lack of flexibility in the power sector—both in terms of supply and demand. As shown in Figure 7C, Shandong’s “duck curve”^96^ remains relatively flat—particularly when compared to places such as California, where wind and solar account for a much larger share of generation but negative prices are rare.

##### Reductions in I&C electricity prices primarily result from T&D efficiencies

**Figure 8:**
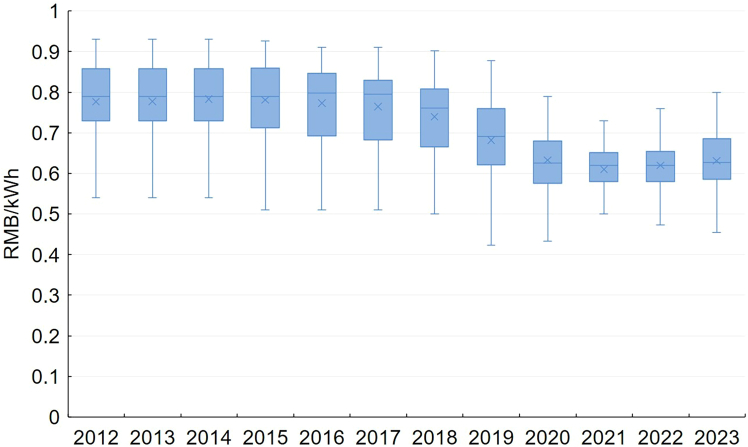
Average tariff for general industrial electricity customers (35 kV and above) in 36 major cities *Source*: Price Monitoring Center, NDRC.

Theoretically, market-oriented reforms are expected to enhance competition on the generation side and reduce electricity prices.^39^^,^^73^ Overall, I&C prices fell significantly while residential prices did not change much.^78^ That is, price reductions mostly benefited non-households, consistent with the Chinese government’s focus on reducing costs to businesses to foster economic growth.^37^ Some studies of electricity prices in Jiangsu, Guangdong, and Zhejiang found that nominal I&C electricity prices decreased by 20–30% since January 2012.^91^^,^^97^ Figure 8 illustrates the decline in industrial electricity prices in 36 major cities from 2012 to 2023. Most studies find that these pricing decreases do not come primarily from improved market efficiency, increased competition, or cost reductions, but rather from the government’s use of administrative means to reduce electricity prices.^91^ This is partly similar to the experience in the United States, where part of the post-restructuring price decline was tied to government commitments made during the reform process. However, the U.S. electricity market is more mature, with a stronger link between underlying costs and end-user prices. As a result, much of the U.S. price variability can also be attributed to technological advances in generation and volatility in natural gas prices.^28^

Xie et al. (2020)^91^ conducted a study on Guangdong and Zhejiang provinces, the pioneers of electricity market reforms, finding that the majority of post-reform price reductions were driven by lower T&D tariffs. In fact, the Chinese government has explicitly identified the reduction of T&D prices as a primary strategy for lowering electricity costs.^98^ These price reductions have occurred mainly due to the regulation of T&D enterprises, as reform has transformed their “duopoly” as sole buyers and sellers. Prior to reform, grid company revenues came primarily through an energy (volumetric) tariff on the difference between selling and buying prices.^34^ Since reform, T&D tariffs are set based on “rate of return” regulation, aimed at establishing cost-of-service remuneration with some additional performance incentives. On a three-year cycle, expected costs are converted into tariffs based on expected demand.^37^ This shift has dramatically reduced T&D tariffs. Xie et al.^99^ analyzed the impact of the 2015 reform on 30 of China’s provincial grid companies, finding that T&D tariff reforms and other changes in regulatory mechanisms had a direct negative impact on profitability.

##### Time-of-use pricing has improved the social benefit

To optimize power resource allocation, China established TOU tariffs for I&C users in the 2015 reforms, encouraging market transactions to set peak/shoulder/off-peak (PSO) price ratios. Guo et al.^55^ examine the effectiveness of mandatory TOU pricing for I&C users in Guangdong. They reveal that the social benefits of TOU tariffs can reach up to approximately 4.5% of wholesale electricity generation costs, with nearly 98% of these benefits arising from savings in capacity investment. However, the current PSO price ratio is not optimal. Widening the price gap between peak and off-peak periods can substantially enhance social benefits. The authors demonstrate that social benefits increase by approximately 70% at the optimal price ratio, indicating that social benefits of China’s TOU tariffs could rise to 7.6% of wholesale electricity generation costs. This substantial improvement is driven by several factors. First, current off-peak demand remains underutilized; widening the price gap can incentivize users to shift production activities to off-peak hours, lowering their operating costs. Second, encouraging consumers to shift non-essential electricity use to low-load periods enhances resource utilization, reduces reliance on high-cost generators and ancillary services, and cuts fuel and maintenance expenses. Third, a larger peak-to-off-peak price difference helps alleviate peak demand, reducing the need for costly infrastructure investments designed to handle peak loads.

#### Market integration enhances efficiency and welfare but raises equity concerns

**Figure 9:**
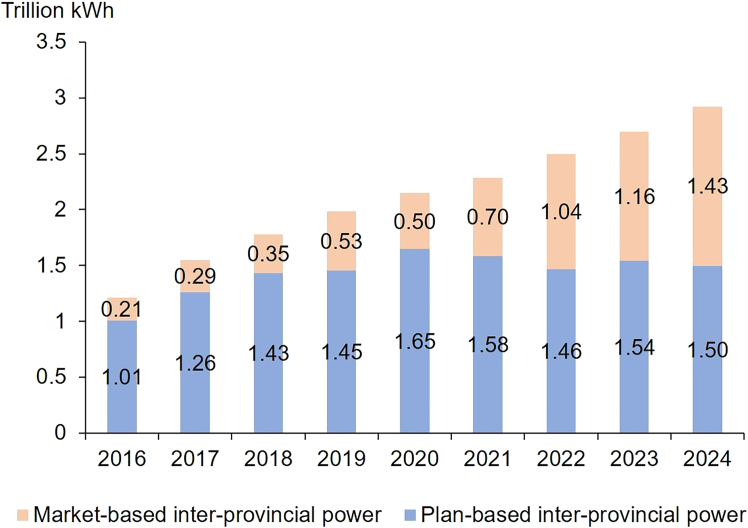
Share of inter-provincial power based on markets and government plans

In January 2022, the central government issued an important document announcing its intention to establish a unified national electricity market by 2030.^64^ Prior to 2015, market-based electricity trading between provinces was minimal, with most cross-provincial and cross-regional electricity carried out under long-term government agreements. Since the 2015 reforms, inter-provincial electricity trading has gradually increased. By 2024, nearly 50% of cross-provincial and cross-regional electricity flows were conducted through competitive market mechanisms. Figure 9 depicts the scale of inter-provincial electricity transactions in China.

With the national electricity market yet to be fully unified, current research mainly uses scenario-based simulations to assess the potential effects of market integration. Research has predominantly focused on southern China, where integration among the five provinces has advanced more rapidly. Findings suggest that market integration can optimize generation capacity, boost production efficiency, lower wholesale electricity prices, and enhance social welfare. Chen et al.^100^ estimated that consolidating southern China’s provincial markets into a regional market could reduce electricity prices, cut carbon emissions, and generate seven billion yuan in additional social benefits. Similarly, Abhyankar et al.^76^ estimated that market-based operations in the region led to a 35% reduction in wholesale electricity costs, with 40% of these savings achievable without transmission expansion.

Despite these benefits, societal gains from market integration are unevenly distributed across provinces and stakeholders, potentially leading to equity concerns among regions and between producers and consumers. In examining implications of establishing a unified national electricity market, Chen et al.^100^ find that market integration can enhance inter-provincial electricity trade and drive price convergence across regions. This process could result in higher electricity prices in provinces that primarily export electricity, while reducing prices in those that rely on imports, thereby reshaping regional price dynamics. The resulting higher electricity prices in some exporting provinces could negatively affect local consumers, while generators in resource-poor eastern provinces may face losses as production shifts to resource-abundant western regions.

### Future challenges of China’s electricity market reform

#### Challenges in balancing the roles of government and markets

State control and marketization are not mutually exclusive but rather complementary processes.^33^ While advancing the role of the market is a key goal of China’s electricity reform, that does not entail a simple withdrawal of the state. Over time, the state’s role must evolve from direct control of operations and pricing to more strategic institutional responsibilities, such as incentivizing regulation, addressing market power, coordinating activities, and engaging in long-term planning.^8^^,^^11^^,^^30^^,^^101^^,^^102^ That markets and regulation are both imperfect necessitates finding an optimal balance between them.^103^ Achieving this balance is no easy task, as calibrating the extent of government involvement in market operations remains a delicate challenge. China is currently navigating this transitional phase, which has brought forth a range of complexities and challenges as discussed in this section.

##### How to balance cost recovery and price volatility in regulating electricity prices?

Electricity prices in China are not fully market-driven. Rather, China’s power sector operates under a “dual-track” system, combining both planned and market-based mechanisms. In the planned track, the grid purchases electricity from priority generators and supplies it to priority users, with feed-in and sales prices determined by the government. Priority generating units include renewable energy units, units planned for inter-provincial power supply, and cogeneration units guaranteeing heat supply. Priority consumers include agriculture, residents, and important public entities. Even within the wholesale market, the government regulates prices through mechanisms such as upper and lower price caps in both spot and long-term markets. These caps aim to mitigate excessive price volatility, shielding market participants while effectively constraining market forces.

Determining how best to adjust benchmark prices and price fluctuation ranges in line with cost changes poses a formidable challenge, one that demands a high degree of regulatory sophistication. Recent developments suggest that existing price benchmarks and fluctuation ranges might require recalibration. For instance, since 2019, the benchmark price for coal-fired power in the long-term market has been based on a coal price of 535 yuan/ton. Despite coal prices surpassing 800 yuan/ton since 2021, this benchmark remains unchanged. This disconnect has left coal-fired generators unable to recover their costs, leading to substantial financial strain.^104^^,^^105^ Even so, eliminating price regulation is unlikely to be feasible in the foreseeable future. However, it is possible to improve market efficiency by adjusting benchmark prices more frequently to reflect changes in fuel costs and by expanding the price bands. The key challenge lies in striking an appropriate balance—designing price caps and benchmarks that capture market dynamics while safeguarding economic efficiency and system security.

##### How to regulate market power on the generation side?

**Figure 10:**
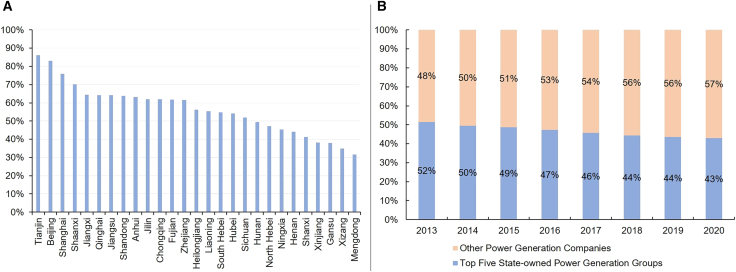
Market structure and concentration in China’s power generation sector (A) Concentration ratio of installed capacity owned by the top four companies (CR4) in provincial markets within State Grid regions, 2023. *Source*: Data from provincial electricity trading centers. (B) Market share of installed capacity held by the five major power generation groups. The “five major power generation groups” denote the leading central state-owned enterprises in power generation, formed during the 2002 restructuring. *Source*: Enterprises’ corporate social responsibility reports and industry annual statistical compilations.

China’s power generation sector remains highly concentrated. To date, its market structure is still primarily shaped by the horizontal unbundling reforms of 2002, with the 2015 reforms having little impact on market concentration. Most provincial markets exhibit considerable oligopoly dominance. In 2023, the concentration ratio of the top four companies (CR4) in State Grid provincial markets exceeded 30% in all provinces, with 18 provinces surpassing 50%, and the highest reaching 86.7% (see Figure 10A). Nationally, the market share of the five major state-owned power generation groups has been declining, dropping from 52% in 2013 to 43% in 2020, but it remains substantial (see Figure 10B). In 2020, due to operational difficulties, coal-fired power plants owned by these five groups were re-integrated across five northwestern provinces (Gansu, Shaanxi, Xinjiang, Qinghai, and Ningxia). This restructuring left each province’s coal power sector under the leadership of a single central enterprise. Other generation sources, including coal plants operated by local state-owned and private enterprises, continue to operate independently, preserving the overall competitive framework. Nevertheless, the integration has intensified market power concerns in these provinces.

The vertical unbundling of electric utilities, coupled with strict rate-of-return regulation, has successfully mitigated market power. Although explicit abuses have not been observed, the expansion of the spot market is likely to heighten the risk of such behavior in the power generation sector. Moreover, the fragmentation of China’s electricity market could intensify localized monopolistic tendencies. During peak demand periods, tight electricity supply creates opportunities for power generation companies to exert market power, and decisions by individual generators have a heightened impact on market outcomes during these times. Power generators may either directly bid higher prices or indirectly raise prices by reducing generation output.^106^ Such practices lead to higher electricity costs for consumers and a notable decline in overall consumer welfare.^107^^,^^108^ To mitigate these risks, both competitive and regulatory measures can be explored. From a competitive standpoint, several strategies can help mitigate market power. First, broadening market access for private generators stimulates competition.^29^ Second, advancing regional and national electricity market integration counteracts localized monopolies. Third, implementing more refined trading mechanisms, such as locational marginal price (LMP), prevents generators from leveraging geographical advantages to exert market power.^11^ From a regulatory perspective, enhancing real-time market power surveillance, implementing reasonable price caps, reinforcing antitrust enforcement, and developing a credit rating system could serve as effective safeguards.^109^^,^^110^^,^^111^ Without adequate countermeasures, such risks could materialize as so-called “gray rhino” events, with predictable but consequential impacts.

##### How to advance market integration?

The geographic mismatch between China’s energy resources and electricity demand underscores the need to establish a unified national electricity market. However, two pressing challenges must be addressed. First, the rigidity of inter-provincial electricity trading agreements must be relaxed, as it limits market flexibility. Current inter-provincial agreements often set predetermined export schedules well in advance—sometimes more than a year—leaving little room for adjustments based on real-time demand. This inflexibility undermines the role of market price signals and often results in inefficient outcomes, such as regions with high generation costs exporting electricity at a loss or high-demand areas struggling to procure power economically. For instance, in the Southern Power Grid, Yunnan’s hydropower exports to Guangdong have been strained by seasonal resource variability and evolving regional electricity needs. Similarly, Shandong’s rapid solar expansion has resulted in frequent midday power surpluses, reducing its willingness to import electricity at those times. However, during evening peaks, Shandong often depends on imports, which can conflict with supply shortages in exporting regions, causing reluctance from exporting provinces to send power at previously agreed prices. Expediting the development of inter-provincial day-ahead and real-time electricity markets could significantly enhance market flexibility and better align supply with demand.^40^

Second, other inter-provincial barriers must be addressed.^112^ While the 2015 reforms encouraged the development of provincial electricity markets, differences in trading models, products, and regulatory frameworks across provinces have complicated efforts to integrate market operations. Moreover, local governments often exhibit protectionist tendencies. Market integration optimizes resource allocation on a broader scale but inevitably redistributes benefits. Some regions or enterprises may lose advantages under an integrated market, leading to resistance against reforms. Building a unified electricity market, therefore depends on balancing the interests of different stakeholders, whether through market design or government intervention. In the short term, targeted fiscal transfers and capacity compensation may ease opposition from certain market players. However, in the long run, real-time dynamic pricing is expected to become the norm, benefiting all stakeholders.^113^

##### How to reform residential electricity pricing?

China’s electricity reforms have historically focused on I&C users, with minimal attention given to residential consumers. This pattern is not unique to China. In fact, most reforms start by liberalizing I&C tariffs globally. For example, in the United States, even two decades after restructuring its power sector, residential electricity prices remain largely under regulatory control.^28^ Currently, residential and agricultural users benefit from cross-subsidies funded by higher electricity prices for I&C users, giving them relatively low rates.^114^ While this approach treats electricity as a basic public good, research suggests that such subsidy mechanisms may impose excessive financial burdens on I&C users and distort market price signals.^115^ Another question is whether electricity prices should be the same for the wealthy and the poor. To address this, China has implemented tiered electricity pricing for households, where higher consumption is charged at progressively higher rates. This policy aims to encourage energy conservation among residents through price incentives while maintaining fairness.^116^

Future reforms may need to place greater emphasis on residential consumers. From an equity standpoint, more refined pricing mechanisms may be necessary to consider disparities between rural and urban households, as well as the economic divide between China’s developed eastern provinces and less-developed western regions.^114^ From an efficiency perspective, residential users are likely to be increasingly integrated into electricity markets, and cross-subsidies from I&C users progressively reduced. This transition could raise electricity prices, posing the challenge of introducing market mechanisms while protecting vulnerable consumers. Moreover, integrating residential consumers into electricity markets may be constrained by the need for extensive smart meter deployment. To address this challenge, some studies propose that ISOs could adopt statistical sampling techniques for aggregations of consumers, rather than relying on the direct measurement of each individual load. This approach could facilitate residential market participation even in scenarios where smart meter penetration remains limited.^117^

#### Challenges in ensuring flexibility and adequacy in China’s power system

The growing share of renewable energy poses significant challenges to China’s electricity market design due to the inherent intermittency and variability of renewable resources. The primary concern lies in ensuring flexibility and long-term adequacy of electricity system. In theory, a well-structured electricity market can address these challenges effectively. However, in practice, additional policy objectives, such as climate goals and economic development, introduce complexities—particularly during the transitional phase of electricity market reforms.

##### How to ensure flexibility?

Ensuring power system flexibility is a critical issue in the energy transition. As renewable energy penetration increases, power systems become more vulnerable to natural variability, increasing the risk of supply shortages. This necessitates timely generation from flexible units such as thermal power plants and energy storage systems to maintain system balance. Additionally, the variable nature of renewables places greater demands on grid stability, particularly for frequency and voltage control, emphasizing the critical role of ancillary services provided by flexible resources.^35^^,^^63^^,^^118^ Experiences from regions with high levels of renewable energy penetration—such as Europe and California—suggest that flexibility challenges can be addressed, at least to some extent, through well-designed market mechanisms and a diverse portfolio of flexible resources. These include dispatchable conventional generators, grid-scale storage, demand-side response, and interregional electricity trading. In California, for instance, a combination of emerging battery storage, interregional exchanges, natural gas and hydropower generation, and the Flexible Ramping Product (FRP)—a market instrument specifically designed to handle variability—has proven adequate to manage a system where wind and solar now contribute around 40% of total generation. While China’s power system is structurally different—being heavily reliant on coal, which is less flexible than gas-fired power. However, experiences from countries such as Germany and Denmark, as well as recent domestic developments, show that there are numerous technical options to enhance the operational flexibility of coal-fired plants. Moreover, large-scale battery storage has played an increasingly prominent role in improving grid responsiveness. These insights underscore the importance of maintaining a technology-neutral approach, allowing all resources that can reliably provide system flexibility to compete on equal footing within the market.^27^^,^^48^^,^^119^

In the future, China’s market-oriented policies may require further refinement to fully unlock their potential. Key strategies include incentivizing energy storage participation in spot and ancillary service markets^53^; encouraging demand-side flexibility through expanded TOU pricing differentials; reforming capacity payments to adopt technology-neutral and performance-based compensation frameworks that promote fair competition between traditional providers (e.g., coal-fired power plants) and emerging flexibility providers (e.g., battery storage and demand-side aggregators); and establishing a unified national market to enhance signals for energy availability and scarcity on a broader scale.^48^^,^^120^

##### How to ensure adequacy?

Ensuring the long-term adequacy of power systems is another critical challenge. This issue is largely attributed to the “missing money” problem: revenues from energy and ancillary service markets are often insufficient to incentivize needed investment in new generation capacity.^121^ Price caps further compound this challenge by limiting generators’ ability to earn scarcity rents during supply shortages. High renewable penetrations exacerbate the missing money problem because increased entry of variable energy resources with near-zero marginal costs decreases the amount of thermal generation cleared from the merit-order stack and induces lower capacity factors for thermal generators. In addition, energy prices tend to be lower (on average) and more volatile as a result of increasing renewable power participation.^122^

Traditional approaches to addressing the missing money problem include several key mechanisms. Scarcity pricing is one such solution, allowing peak prices to reflect capacity scarcity more accurately.^123^ Capacity mechanisms, including fixed payments or capacity markets, are another common approach. Contracts for difference (CFD) offer an additional solution, providing revenue stability for generators while incentivizing availability during scarcity conditions. These contracts also protect consumers from extreme price volatility, as system operators can procure electricity at pre-specified prices.^124^

Several recent studies introduce novel measures for enhancing long-term adequacy in power systems. Prete et al.^125^ categorize 11 such approaches into four areas. The first, aimed at capacity markets, introduces a joint procurement mechanism for forward capacity and clean energy attribute credits and represents the least radical departure from current market structures.^126^ The second area focuses on long-term contracts, aiming to ensure revenue certainty through fixed-price agreements.^127^^,^^128^^,^^129^ The third targets short-term markets, proposing the separation of renewable and non-renewable electricity markets, with operational rules tailored to the specific characteristics of different generation technologies.^130^ The fourth approach introduces a “swing-contract” market design and would require a comprehensive structural overhaul of China’s electricity market. Under this mechanism, dispatchable resources—including generators and energy storage systems—can compete on equal terms to provide flexible power paths. By enabling the trading of reserve, swing contracts help ensure adequate real-time balancing capabilities in future system operations.^131^ Whether relying on traditional mechanisms or adopting innovative approaches, significant theoretical and empirical exploration is still needed to evaluate their viability in addressing China’s long-term electricity adequacy challenges as it moves toward a carbon-neutral power system.

## Discussion

China’s electricity sector reforms since 2015 mark a pivotal shift toward harnessing market-oriented mechanisms to balance efficiency, equity, and sustainability in the world’s largest power system. The reform agenda has centered on fostering competition in generation and retail, tightening regulatory oversight of grid costs and services, integrating regional electricity markets, and accelerating the transition to renewable energy. Significant progress has been made in multiple areas. First, a comprehensive wholesale market structure has been established, with medium- and long-term contracts forming the backbone, complemented by spot markets, ancillary service markets, and capacity compensation schemes. Asa result, the share of market-based electricity transactions nearly quintupled in eight years, increasing from 13% in 2015 to over 61% in 2023. Second, on the consumer side, I&C users now have the option to engage in direct power purchase agreements with generators or access competitive pricing through retail markets, meaning that grid companies are no longer the sole channel for electricity procurement. Third, enhanced regulation of T&D networks has led to the introduction of a transparent pricing framework, significant cost reductions, and greater private sector participation. Finally, wind and solar power projects have increasingly shifted toward market-based trading mechanisms supported by contracts for difference (CFD).

Empirical evidence suggests that shifting from fairness-based dispatch to economic dispatch has significantly enhanced operational efficiency, with coal-fired power plants achieving efficiency gains of 3–15%.^4^^,^^72^^,^^73^^,^^75^^,^^76^^,^^77^^,^^132^ In the short term, this effect has been particularly pronounced for smaller-capacity generators due to stronger competitive pressure. Over the longer term, however, due to persistently high marginal costs that are difficult to offset through efficiency improvements alone, marketization may lead to the gradual market exit of these units. Their continued viability will likely depend on retrofitting for enhanced operational flexibility under high renewable penetration.

However, China’s electricity market transition remains incomplete. Despite the introduction of market-oriented mechanisms, electricity pricing continues to be heavily regulated, preventing cost-reflective pricing. The observed decline in I&C electricity prices is largely attributed to strict government regulation in T&D tariffs rather than increased market competition.^91^ While spot markets effectively capture supply and demand fluctuations, their limited trading volumes have restricted the extent to which costs and supply-demand conditions are reflected in electricity prices. Greater market integration has fostered inter-provincial electricity trade, boosting overall efficiency and social welfare. However, despite these benefits, the distribution of welfare gains from market integration has been uneven across provinces and stakeholders, potentially creating substantial barriers to the advancement of a more integrated electricity market.

Looking ahead, China’s power market reform must navigate two fundamental challenges: balancing government intervention with market dynamics and ensuring both system flexibility and long-term adequacy. The first challenge lies in defining the government’s role in an increasingly market-driven power sector. While marketization remains a core objective, government intervention is still crucial for managing market power and protecting vulnerable consumers. Currently, electricity prices are still subject to partial regulation, yet delayed adjustments in benchmark tariffs and restrictive price caps hinder cost-reflective pricing. The concentrated market power within the generation sector further complicates competition, underscoring the need to enhance market access and strengthen regulatory oversight. Furthermore, inter-provincial electricity trade remains constrained by rigid agreements and local protectionism, reducing market efficiency. Expanding flexible trading mechanisms and harmonizing market rules across provinces would be effective measures for market integration. Residential electricity pricing is another area requiring policy reform, with a key consideration being how to introduce market mechanisms while protecting the most vulnerable consumers. Achieving the right equilibrium between government regulation and market autonomy will be crucial for sustaining reform momentum.

The second challenge is ensuring system flexibility and long-term adequacy, particularly as renewable energy penetration increases. The inherent variability of renewables demands a more adaptive power grid, along with enhanced market mechanisms for ancillary services, energy storage, and demand-side flexibility. Meanwhile, the issue of “missing money” threatens investment in new generation capacity, as price caps and the low market-clearing prices induced by renewables undermine generators’ long-term profitability. Addressing these issues requires comprehensive market reforms, with potential measures including improved scarcity pricing, capacity mechanisms, long-term markets, and contracts for difference.^125^ However, substantial theoretical and empirical research is still needed to assess the feasibility of these measures in addressing China’s long-term power adequacy challenges.

Enhancing market efficiency, strengthening system reliability, and advancing renewable energy integration remain central to China’s electricity market reform, necessitating ongoing refinement and adaptation. Despite substantial achievements, market mechanisms are still evolving amid a pivotal energy transition. Further research is essential for defining the optimal trajectory for China’s electricity market development. Ultimately, the success of these reforms will extend far beyond the power sector, influencing China’s economic growth, energy security, and carbon neutrality goals.

### Limitations of the study

(1) Limited comparative analysis. Although this study includes some comparisons between China’s electricity market and international experiences, as well as across provincial markets within China, a more comprehensive and systematic comparative analysis could not be conducted due to space limitations. Future work could deepen these comparisons, providing more context-specific insights to guide reform and offering more generalizable lessons for international power market transitions.
(2) Data availability constraints. Due to the lack of post-2018 operational data at the plant level, we were unable to explore the longer-term effects of market reform on the power generation sector. In particular, the reform’s influence on plant investment and retirement decisions remains an important yet unexamined dimension.
(3) Unquantified impact on renewables. Data limitations also prevented us from quantifying the reform’s effects on renewable energy sources such as wind and solar. Given the distinct technical characteristics and policy environments of different generation technologies, future research is needed to assess how market reforms differentially affect renewables compared to coal-fired generation.

## Resource availability

### Lead contact

Further information and requests should be directed to the lead contact and corresponding author, Jiang Lin (lin.jiang@berkeley.edu).

### Materials availability

The study did not generate new materials.

### Data and code availability

• All data reported in this article will be shared by the lead contact upon request.
• This article does not report original code.
• Any additional information required to reanalyze the data reported in this article is available from the lead contact upon request.

## Acknowledgments

Authors acknowledge the 10.13039/501100001809National Natural Science Foundation of China (grant 72141308).

## Author contributions

Y.F., J.L., and F.S. contributed to the discussion and framework of the article content. Y.F. designed the initial article. J.L., F.S., and M.H. provided substantial feedback and revisions to improve the article.

## Declaration of interests

The authors declare no competing interests.

## STAR★Methods

### Method details

#### Regression of generation efficiency and reform

To further test this relationship between coal intensity, reform, and installed capacity, we estimate the following regression on plant-level data for all coal power plants in the country from 2012 to 2017:

(Equation 1) $${Intensity}_{it}=\beta_{1}{Reform}_{t}+\beta_{2}{Capacity}_{it}+\beta_{3}{Reform}_{t}\times {Capacity}_{it}+\lambda_{p}+\lambda_{y}+\varepsilon_{it}$$

Where *Intensity*_*it*_ and *Capacity*_*it*_ are the log coal intensity and log installed capacity of plant *i* in year *t*; *Reform*_*t*_ represents whether reforms are implemented at year *t*. We divided the whole sample into two periods: for 2015 and before, *Reform*_*t*_ is 0, and after 2015, *Reform*_*t*_ is 1; λ_y_ is the year effect that does not change among individual plants; λ_p_ is the provincial effect that does not change with time; and ε_it_ is the independent error term.

#### Regression of end-user electricity price and coal price

To test the relationship between coal prices, To test the relationship between coal prices, reform, and end-user prices, we estimate the following regression on province-level monthly changes in end-user prices and coal prices.

(Equation 2) $$\begin{array}{c} \Delta E{lec_{price}}_{it}=\beta_{1}{Reform}_{i,t}+\beta_{2}\Delta Coal{\_price}_{i,t}+{\beta_{3}Reform}_{i,t}\times \Delta Coal{\_price}_{i,t}+X_{it}+\varepsilon_{it} \end{array}$$

Where $\Delta E{lec\_price}_{it}=\ln\left(E{lec\_price}_{i,t}\right)-\ln\left(E{lec\_price}_{i,t-1}\right)$ and $\Delta Coal{\_price}_{it}=\ln\left(Coal{\_price}_{i,t}\right)-\ln\left(Coal{\_price}_{i,t-1}\right)$ are the monthly changes in log province average electricity rates and log province coal prices, respectively. We use 2014 as our base year (to capture any changes during 2015) and compare it to 2020. $X_{it}$ denotes the set of control variables, which include city-level per capita GDP, the city-level share of secondary industry, the provincial share of renewable energy generation, and the installed capacity of thermal power in the provincial. $\varepsilon_{it}$ is the independent error term.

#### Regression of spot market prices and coal price

Taking the Shandong spot market as an example, December Taking the Shandong spot market as an example, we use data from December 2021 to April 2024 and sum 15-minute interval data to daily data for the regression as shown in Equation 3:

(Equation 3) $${Spot\_price}_{t}={\beta_{1}Coal\_price}_{t}+{\beta_{2}demand}_{t}+\lambda_{m}+\delta_{y}+\varepsilon_{t}$$

Where $Spot{\_price}_{t}$ is the log average spot price on day t, including both day-ahead and real-time prices; $Coal{\_price}_{t}$ represents the log coal price on day t, which is derived from the price of 5200 kcal thermal coal in Jining; ${demand}_{t}$ represents the log average net load to participate in the spot market every 15 minutes on day t, defined as total electricity load minus wind and solar generation during each interval; $\lambda_{m}$ is the month fixed effect; $\delta_{y}$ is the year fixed effect; and $\varepsilon_{t}$ is the independent error term.

### Quantification and statistical analysis

We conducted all regressions using Stata. For the regression of generation efficiency and reform, the power plant dataset comes from China Electricity Council, which is the national association for the electricity industry founded with the approval of the State Council. Our dataset covers more than 10,000 power plants from 2012 to 2017, with detailed information on plant-level annual electricity generation, operating hours, generator capacities, coal intensity, coal consumption, and names of plants. All power plants with installed capacity of 6 MW or higher are covered by this dataset. Since this is quite a low threshold for coal-fired power plants, this dataset covers nearly all coal plants in China. For instance, in 2012, the total installed capacity for plants above 6 MW is 754 GW, accounting for 99.86% of the country’s total 755 GW coal capacity. For the regression of end-user electricity price and coal price, monthly coal prices for each province are obtained from the Price Monitoring Center of the National Development and Reform Commission, and electricity price data, from the same source, are monthly prices for industrial customers (35 kV and above) in 36 major cities. These data can be obtained from the CEIC database (https://www.ceicdata.com/zh-hans). For the regression of spot market prices and coal price, data are obtained from Dianchacha (https://dianchacha.cn/home/).

For all other figures, tables, or text content, the data sources are clearly indicated either in the figure legends, table legends, or reference list. All statistical details (standard errors and *p*-values) are provided in the corresponding table legends.
